# Inhibitory Effect of Astaxanthin on Gene Expression Changes in *Helicobacter pylori*-Infected Human Gastric Epithelial Cells

**DOI:** 10.3390/nu13124281

**Published:** 2021-11-27

**Authors:** Suhn Hyung Kim, Hyeyoung Kim

**Affiliations:** Department of Food and Nutrition, College of Human Ecology, Yonsei University, Seoul 03722, Korea; cigdoli2@naver.com

**Keywords:** *Helicobacter pylori*, gastric epithelial cells, astaxanthin, β-catenin, cell proliferation, gastric cancer

## Abstract

*Helicobacter pylori* (*H. pylori*) infection promotes gastric carcinogenesis by increasing oxidative stress, inflammation, and dysregulation of cell survival and proliferation of gastric epithelial cells. Astaxanthin (ASTX), a bioactive carotenoid, exhibits antioxidant and anticancer effects by modulating aberrant signaling pathways that lead to dysregulation of cell death and proliferation. To elucidate the molecular mechanism of *H. pylori*-induced gastric carcinogenesis and to examine the inhibitory effect of ASTX on *H. pylori*-induced gastric epithelial cell gene expression changes, we performed comparative RNA-sequencing (RNA-Seq) analysis for *H. pylori*-infected gastric epithelial cells treated with or without ASTX. RNA-Seq results reveal that differentially expressed genes (DEGs) in *H. pylori*-infected cells were mainly associated with the Wnt/β-catenin signaling pathway, which is related to cell proliferation. ASTX significantly reversed *H. pylori*-induced transcriptional alterations of the key mediators involved in β-catenin signaling, notably, porcupine (gene symbol, *PORCN)*, spermine oxidase (*SMOX*), bone morphogenetic protein (BMP) and activin membrane-bound inhibitor (*BAMBI*), SMAD family member 4 (*SMAD4*), transforming growth factor-β1 (*TGFB1)*, Fos-like 1 (*FOSLI)*, and c-myc (*MYC)*. We suggest that ASTX may be a potential therapeutic agent that can suppress *H. pylori*-induced proliferation-associated gene expression changes, in part, by counter-regulating the Wnt/β-catenin signaling pathway.

## 1. Introduction

Gastric cancer is the third leading cause of cancer-related mortality worldwide [1]. *Helicobacter pylori* (*H*. *pylori*) infection is a major risk factor for the development of gastric cancer [2]. *H. pylori* infection can promote gastric carcinogenesis by elevated oxidative stress, inflammation, apoptosis, and autophagy and dysregulation of cell survival and proliferation [3]. *H. pylori* virulence factors such as cytotoxin-associated protein (cag A) and vacuolization cytotoxin (vac A) play a major role in the pathogenesis of gastroduodenal disease. However, the molecular signaling network leading to *H. pylori* infection-induced gastric carcinoma is not fully elucidated. To date, several *H. pylori*-triggered oncogenic signaling pathways have been identified—namely, epidermal growth factor receptor (EGFR), β-catenin, activator protein 1 (AP-1), and nuclear factor kappa-light-chain-enhancer of activated B cells (NF-κB) [4,5]. Increased proliferation of gastric epithelial cells is a hallmark feature in gastric carcinogenesis, and the Wnt/β-catenin signaling pathway is the most prominent axis associated with *H. pylori*-induced hyper-proliferation [5].

The evolutionarily conserved Wnt/β-catenin signaling controls embryo development and tissue homeostasis. However, aberrant mutations affecting the Wnt/β-catenin signaling pathway often lead to dysregulation of homeostasis and development of cancers. Under basal conditions, cytosolic β-catenin is constantly degraded by the destruction complex AXIN-adenomatous polyposis coli gene (APC)-glycogen synthase β (GSK3β) that targets β-catenin for proteasomal degradation [6]. However, when Wnt ligand is activated, the Wnt-Frizzled-lipoprotein related protein (LRP) receptor complex inhibits phosphorylation of β-catenin by the destruction complex. Consequently, cytosolic β-catenin is stabilized to stimulate the transcription of its target genes in the nucleus [7].

Aberrant expression or activation of β-catenin and its modulators are frequently observed in gastric cancers, and *H. pylori* infection can upregulate β-catenin signaling to increase gastric epithelial cell proliferation and gastric cancer cell migration [8]. To date, the potential therapeutic and preventive benefits of several natural bioactive compounds have been extensively studied as prospective tumor suppressors for gastric cancer. Previously, we showed that carotenoids β-carotene and astaxanthin (ASTX) inhibit *H. pylori*-induced expression of β-catenin target oncogenes c-myc and cyclins (cyclin E or cyclin D1) in gastric epithelial cells in vitro [9] and in gastric mucosal tissues in vivo [10].

ASTX is a xanthophyll carotenoid abundantly present in red-colored marine animals, such as lobsters, salmon, and krill. ASTX is mostly derived from natural sources, specifically the algae *Haematococcus pluvialis*, salmon, and krill, as an ester of fatty acids [11]. ASTX consists of multiple conjugated double bonds and, therefore, exhibits a powerful antioxidant capacity [12]. Due to the antioxidative property, application of ASTX is significant, ranging from its use in aquaculture to foods to nutraceuticals [13]. Based on its diverse antioxidative properties as well as its direct regulatory function in molecular transcription and activation, ASTX has been demonstrated to be an effective bioactive agent for suppressing cancer cell proliferation and migration [14,15,16].

The current study was carried out to elucidate the cellular signaling induced by *H. pylori* infection, which could turn on the oncogenic machinery in host cells. Furthermore, we evaluated the regulatory potential of ASTX on the *H. pylori*-induced oncogenic pathways and identified the key mediators associated with its inhibitory effect. RNA-sequencing (RNA-Seq) analysis of *H. pylori*-infected human gastric epithelial AGS cells was performed to identify genes that were altered in response to *H. pylori* stimulation and ASTX pretreatment. Herein, we propose a signaling network through which ASTX might counteract the effects of *H. pylori* infection—namely, *H. pylori*-induced gastric hyper-proliferation.

## 2. Materials and Methods

### 2.1. Cell Line and Culture Conditions

The human gastric epithelial cell line AGS (ATCC CRL-1739) was purchased from the American Type Culture Collection (ATCC; Rockville, MD, USA). AGS cells were grown in RPMI 1640 medium (Gibco, Grand Island, NY, USA) supplemented with 10% fetal bovine serum (FBS; Gibco), 100 U/mL penicillin, and 100 μg/mL streptomycin. The cells were cultured at 37 °C in a humidified CO_2_ incubator with 95% air and 5% CO_2_.

### 2.2. Bacterial Strain

*H. pylori* strain NCTC 11637 was obtained from ATCC, and the bacterial cells were grown on chocolate agar plates (Becton-Dickinson Microbiology Systems, Franklin Lakes, NJ, USA) at 37 °C under microaerophilic conditions using an anaerobic chamber (BBL Campy Pouch^®^ System, Becton-Dickinson Microbiology Systems). *H. pylori* was incubated overnight in a humidified CO_2_ incubator before inoculation. Subsequently, *H. pylori* was collected from the plates, suspended in antibiotic-free RPMI 1640 medium supplemented with 10% FBS, and added to the cell culture at a cell/bacterium ratio of 1:100.

### 2.3. Treatment of AGS Cells with ASTX and H. pylori Infection

ASTX (Sigma-Aldrich, St. Louis, MO, USA) was dissolved in dimethyl sulfoxide (DMSO; Sigma-Aldrich, St. Louis, MO, USA) and stored under nitrogen gas at −80 °C. AGS cells (at 80% confluence) were pretreated with 5 μM ASTX or DMSO at 0.05% final concentration (*v*/*v*) as control vehicle for 3 h prior to co-culture with *H. pylori*. The concentration of ASTX and incubation time were adapted from our previous study, which showed that 5 μM ASTX exhibited antioxidative effects against *H. pylori* stimulation when ASTX was preincubated in AGS cells for 3 h [17]. AGS cells were infected with *H. pylori* at a cell/bacterium ratio of 1:100 for 4 h based on our previous study on *H. pylori*-induced gastric proliferation, where mRNA expression of c-myc and cyclin E was significantly increased starting from the 4 h culture time point [18]. Gene expression of *H. pylori*-infected cells (HP) was compared with that of uninfected AGS cells (None) and *H. pylori*-infected cells pretreated with ASTX (ASTX + HP). For the ASTX group, AGS cells were treated with ASTX for 7 h.

### 2.4. Preparation of Total RNA Extracts and Library Construction

Total RNA was extracted using the TRI reagent (Molecular Research Center, Cincinnati, OH, USA) and purified and concentrated using the RNeasy MinElute Cleanup Kit (Qiagen, Valencia, CA, USA) according to the manufacturer’s protocol. Upon determining the total RNA concentration in each extract, the extracts from three replicate experiments were pooled for RNA-Seq library construction. RNA libraries were constructed and compiled using the QuantSeq 3′ mRNA-Seq Library Prep Kit (Lexogen, Vienna, Austria) according to the manufacturer’s instructions. The RNA library was newly generated solely for the current experiment using the aforementioned experimental conditions, disparate from our previous RNA-Seq experiment on ASTX in *H. pylori*-infection [19].

### 2.5. RNA-Sequencing and Bioinformatics Analysis

The total RNA library was subjected to transcriptome sequencing performed by e-Biogen (www.e-biogen.com, Seoul, Korea, accessed on 12 October 2021). QuantSeq 3′ mRNA-Seq reads were aligned using Bowtie2. Bowtie2 indices were either generated from the genome assembly sequence or the representative transcript sequences for aligning to the genome and transcriptome. The alignment file was used for assembling transcripts, estimating their abundances, and detecting differential expression of genes. As QuantSeq focuses on the 3′ end of polyA RNA, only one fragment per transcript is generated and length normalization is not required. Transcript expression values were quantified based on read counts. Differentially expressed genes (DEGs) were determined based on counts of unique and multiple alignments using coverage in Bedtools. The read count data were processed based on the quantile normalization method using edgeR (http://bioconductor.org, accessed on 12 October 2021). A cutoff was applied at a normalized gene expression (log2) of 2.

Excel-based DEG analysis software (ExDEGA; www.e-biogen.com, Seoul, Korea, accessed on 12 October 2021) was used for data mining and data visualization. Genes with >1.5-fold change in transcript levels were considered to be differentially expressed. Gene classification was based on searches performed using DAVID (http://david.abcc.ncifcrf.gov/, accessed on 12 October 2021) and Medline databases (http://www.ncbi.nlm.nih.gov/, accessed on 12 October 2021). To identify functional groups and molecular pathways associated with the observed DEGs, RNA-Seq data were further analyzed using the Kyoto Encyclopedia of Genes and Genomes (KEGG) database (www.genome.jp, accessed on 12 October 2021).

### 2.6. Validation of DEGs Using Real-Time Polymerase Chain Reaction (PCR)

Real-time PCR was performed to validate the expression profiles of key DEGs identified using RNA-Seq analysis. Candidate genes were selected in relation to the category of molecular pathway. Total RNA was converted to cDNA by incubating the RNA sample with a random nucleotide hexamer and MuLV reverse transcriptase (Promega, Madison, WI, USA) at 23 °C for 10 min, 37 °C for 60 min, and then 95 °C for 5 min. Real-time PCR assay was subsequently performed using the cDNA templates and primers specific for human. Sequences of the primers used were 5′-CATCCTCATCTACCTACTCAT-3′ (forward) and 5′-CGCATCTTGTGCCATGTC-3′ (reverse) for porcupine; 5′-CCTACCCTCTCAACGACAGC-3′ (forward) and 5′-TAACTACCTTGGGGGCCTTT-3′ (reverse) for c-myc; 5′-GACCACAATCACGACACTGG-3′ (forward) and 5′-TTAGCACACCTAGCGACACG-3′ (reverse) for spermine oxidase (Smox); 5′-GGCAGCATCACAGTAGCATC-3′ (forward) and 5′-GATCGCCACTCCAGCTACAT-3′ (reverse) for bone morphogenetic protein (BMP) and activating membrane-bound inhibitor (Bambi); and 5′-CATCTGAGTCTAATGCTACC-3′ (forward) and 5′-CAACAGTCCTTCACTATGG-3′ (reverse) for SMAD family member 4 (Smad4). The cDNA was amplified for 40 cycles comprising the following steps: denaturation at 95 °C for 30 s, annealing at 51 °C for 30 s, and extension at 72 °C for 30 s. During the first cycle, the 95 °C step was extended to 3 min. Concurrently, the β-actin gene that served as the reference gene was amplified using the primers 5′-ACCAACTGGGACGACATGGAG-3′ (forward) and 5′-GTGAGGATCTTCATGAGGTAGTC-3′ (reverse). The relative gene expression of target genes was normalized to that of β-actin.

### 2.7. Statistical Analyses

For the changes in the levels of gene expression in *H. pylori*-infected AGS cells, all values are expressed as the mean ± standard error of three samples per group (*n* = 3). Analysis of variance, followed by Neuman–Keuls post hoc test, was used for statistical analyses. Differences were considered statistically significant at *p*-value < 0.05.

## 3. Results

### 3.1. Gene Expression Profile of H. pylori-Infected and/or ASTX-Treated AGS Cells

**Figure 1:**
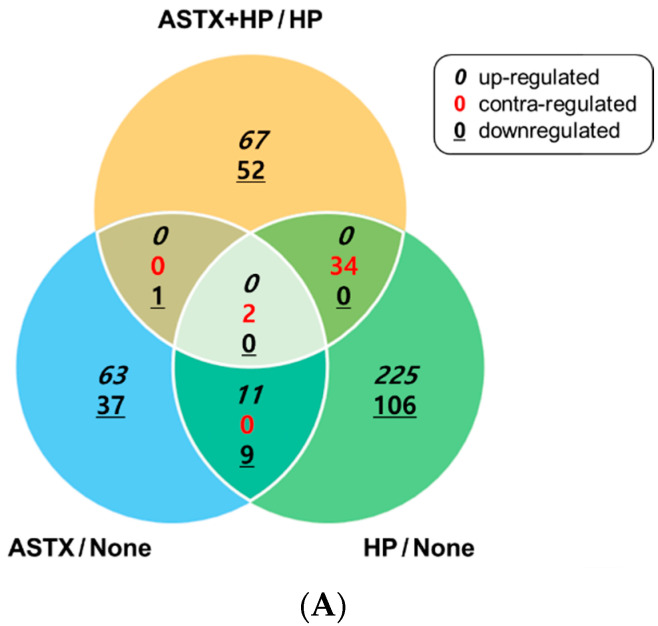

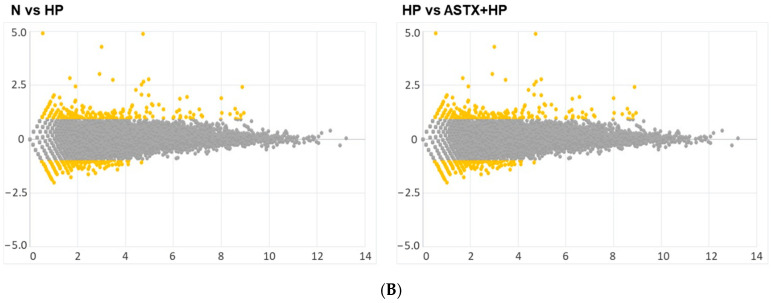
A comparison of differentially expressed gene (DEG) expression pattern observed between pairs, and paired-pairs, of the four experimental groups of uninfected AGS cells (N or None), AGS cells infected with *H. pylori* (HP), AGS cells treated with astaxanthin (ASTX), AGS cells treated with astaxanthin and infected with *H. pylori* (ASTX+HP). (**A**) The Venn diagram summarizes the number of DEGs in each experimental pair. Numbers in black italic font correspond to upregulated DEGs, underlined numbers correspond to downregulated genes, and numbers in red correspond to genes that are upregulated in one set of experimental group and downregulated in the other. (**B**) The MA plots visualize differences in gene intensity value between each experimental pair. The log2 fold changes of all genes were plotted against the mean normalized expression counts. Genes with a fold change >1.5 (shown in yellow) were considered differentially expressed. The y-axis represents log2 fold changes, where positive values (0 < y < 5) indicate upregulated gene expressions and minus values (−5.0 < y < 0) indicate downregulated gene expressions. Without the hyphen: upregruated.

Gene expression profile of *H. pylori*-infected AGS cells (HP) was analyzed and compared with that of uninfected AGS cells (None) and *H. pylori*-infected cells pretreated with ASTX (ASTX + HP). To examine the possible transcriptional effect of ASTX on AGS cells, the gene expression profile of AGS cells treated with ASTX only (ASTX) was also monitored. RNA-Seq analysis on the four experimental groups identified a total of 606 DEGs. The observed DEGs of paired groups are summarized in the Venn diagram and visualized in an MA plot (Figure 1).

To identify the potential mediators of *H. pylori* infection, overlapping DEGs of HP and None groups were filtered out. *H. pylori* upregulated 250 genes and downregulated 136 genes in AGS cells. RNA-Seq results revealed that interleukin 8 (gene symbol, *CXCL8*), chemokine ligand 2 (*CXCL1*), macrophage inflammatory protein 2 (*CXCL2*), *EGFR*, heparin-binding EGF-like growth factor (*HBEGF*), transforming growth factor-β1 (*TGFB1*), Smox (*SMOX*), NF-κB (*NFKB1* and *NFKB2*), TNF-α induced proteins (*TNFAIP2* and *TNFAIP3*), TNF-α induced protein 8 (*TNFAIP8*), porcupine (*PORCN*), S-phase kinase-associated protein 2 (*SKP2*), polycystin2 (*PKD2*), Kruppel-like factors (*KLF5* and *KLF6*), activating transcription factor 3 (*ATF3*), Fos-like 1 (*FOSL1*), and c-myc (*MYC*) genes were upregulated in AGS cells following *H. pylori* infection. Since these genes are associated with inflammation and proliferation, it could be inferred that *H. pylori*-induced inflammation and proliferation may induce carcinogenesis.

To investigate the regulatory effect of ASTX pretreatment on *H. pylori* stimulation, DEGs from the ASTX + HP group were analyzed, of which 88 genes were upregulated and 68 genes were downregulated. Among the genes differentially regulated by ASTX in *H. pylori*-infected cells, insulin-like growth factor 1 receptor (*IGF1R*), gesolin (*GSN*), histone deacetylase 8 (*HDAC8*), signal transducer and activator of transcription 5B (*STAT5B*), Smad4 (*SMAD4*), TGF-β1 (*TGFB1*), Ras oncogene family (*RAB 11FIP3* and *RAB 40C*), S-phase kinase-associated protein 2 (*SKP2*), polycystin2 (*PKD2*), Fos-like 1 (*FOSL1*), and c-myc (*MYC*) genes were notable, suggesting a possible regulatory effect of ASTX on cancer pathways.

### 3.2. Gene Ontology (GO) Annotation and Functional Analysis of DEGs

**Figure 2:**
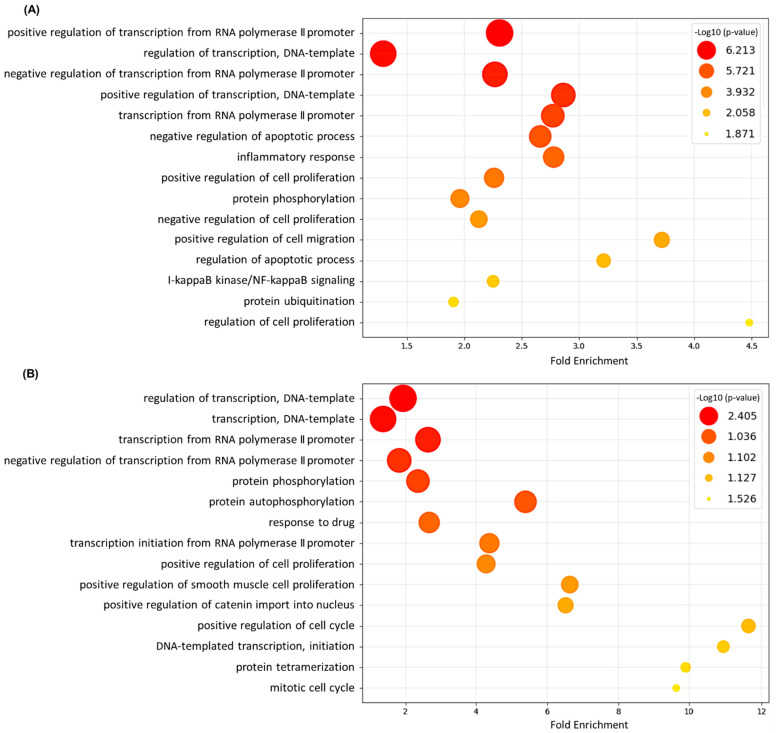
Biological processes in GO enrichment analysis of DEGs. GO enrichment analysis was performed on None vs. HP pair (**A**) and HP vs. ASTX + HP pair (**B**). The color and size of each bubble represents the –Log10 values, and fold enrichment scores are represented on the horizontal axis.

To elucidate the biological processes that may be associated with *H. pylori* stimulation of gastric epithelial cells, DEGs in the first pair (None vs. HP) were aligned according to functional categories using GO annotation. Regulation of transcription (GO:0006355), apoptotic process (GO:0006915), inflammatory response (GO:0006954), and regulation of cell proliferation (GO:0008284, GO:0008285) were the most commonly observed GO annotation terms for biological processes (Figure 2A).

GO functional annotation was then performed on DEGs in the ASTX + HP group. Regulation of transcription (GO:0006355) and regulation of cell proliferation (GO:0008284, GO:0008285) were markedly enriched biological processes in *H. pylori*-infected AGS cells pretreated with ASTX (Figure 2B).

**Figure 3:**
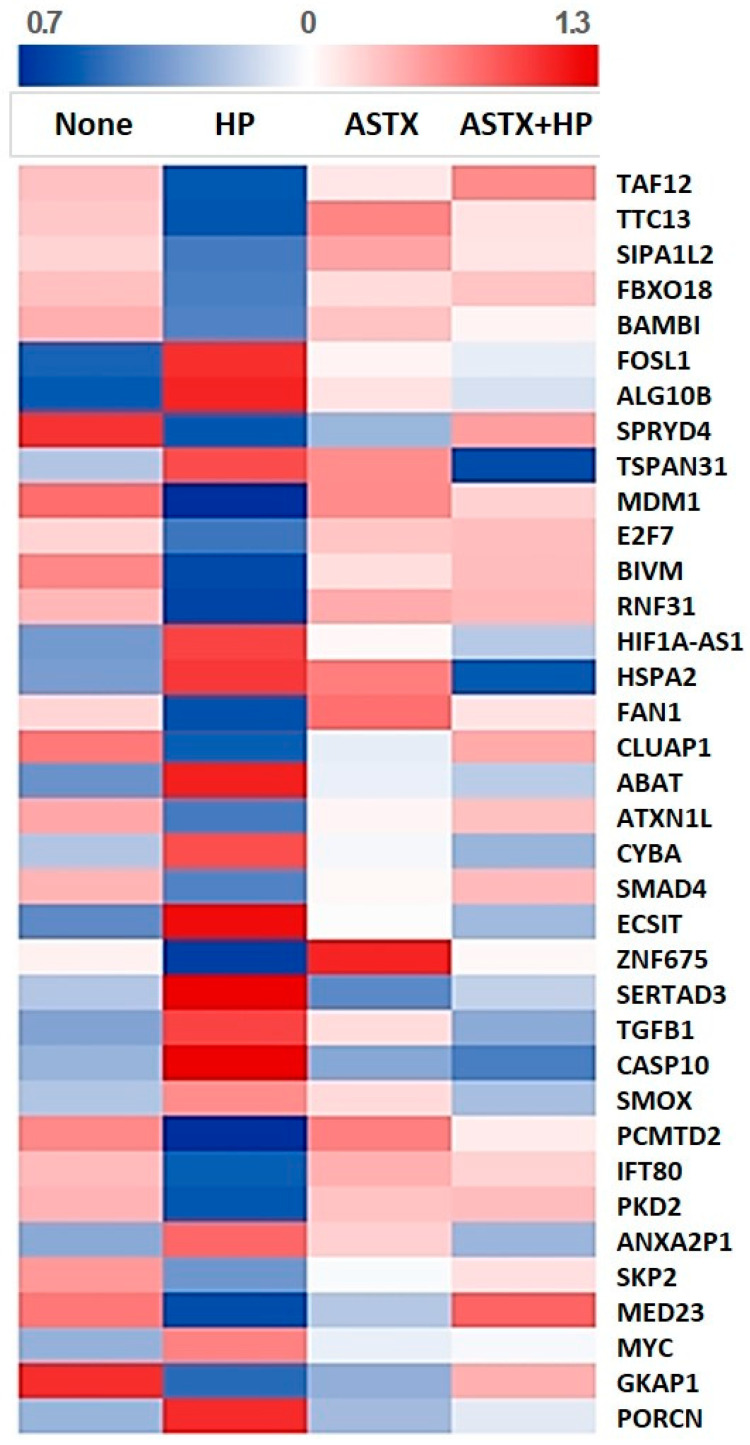
Heatmap representation of the expression levels of candidate DEGs. The color gradient visualizes the fold change values of each gene expression, where blue represents downregulated expression and red represents upregulated expression.

To discover the inhibitory mechanism of ASTX on *H. pylori*-induced transcriptional alterations, expression of genes that were counter-regulated by *H. pylori* and ASTX, either increased by *H. pylori* infection but decreased in ASTX-pretreated cells or vice versa, were chosen as candidate DEGs of particular interest. Figure 3 illustrates a heatmap representing the expression patterns of the candidate DEGs. Pathway analysis of the candidate DEGs showed that the genes are mostly involved in the CTCF and Wnt signaling pathways (BioCarta), as well as pathways in cancer and Wnt signaling pathways (KEGG).

### 3.3. Molecular Pathway Analysis of DEGs Involved in Cell Proliferation

Based on the GO and functional analysis, we narrowed our focus to candidate DEGs that were associated with the regulation of cell proliferation via Wnt/β-catenin signaling. Among the candidate DEGs, Bambi (*BAMBI*), Fos-like 1 (*FOSL1*), tetraspanin 31 (*TSPAN31*), E2F transcription factor 7 (*E2F7*), ataxin 1 like (*ATXN1L*), Smad4 (*SMAD4*), zinc finger protein 675 (*ZNF675*), SERTA domain-containing protein 3 (*SERTAD3*), TGF-β1 (*TGFB1*), Smox (*SMOX*), polycystin2 (*PKD2*), S-phase kinase-associated protein 2 (*SKP2*), c-myc (*MYC*), and porcupine (*PORCN*) were annotated in the Wnt/β-catenin pathway. Table 1 shows the list of DEGs reported to be associated with the Wnt/β-catenin pathway, along with the normalized read count of each gene.

The *PORCN* gene, which encodes the Wnt protein activator porcupine, was upregulated in *H. pylori*-infected cells, but ASTX significantly reduced its expression. Transcriptional expression of Fos-like 1 (*FOSL1*) and c-myc (*MYC*) were also significantly increased by *H. pylori* but suppressed by ASTX. Both Fos-like 1 and c-myc are direct downstream target genes of β-catenin [20]. Our results suggest that the β-catenin pathway was stimulated in our experimental setting and that ASTX treatment may have downregulated the *H. pylori*-induced β-catenin signaling.

Smox is a polyamine oxidase that generates H_2_O_2_ as a byproduct of its catabolic activity. A group of researchers demonstrated that *H. pylori* increased Smox expression, thereby activating β-catenin signaling in gastric epithelial cells, which is in line with our hypothesis [21]. In this study, we found that *SMOX* expression was markedly elevated in *H. pylori*-infected cells and that ASTX treatment significantly decreased the *H. pylori*-induced *SMOX* expression.

By contrast, ASTX treatment reversed *H. pylori*-induced repression of *SMAD4* and *BAMBI*. Bambi belongs to the TGF-β family that regulates cell proliferation and survival [19]. In our experiment, *H. pylori*-infected AGS cells showed decreased expression of *BAMBI* but increased expression of *TGFB1*. Although Bambi is known as a β-catenin responsive gene that is upregulated in gastric cancer [22], loss of Bambi expression has been observed in some other invasive cancer models [23,24]. Smad4 has been reported to be a tumor suppressor in gastric cancer [25], and studies on experimental gastric cancer revealed that Smad4 could be downregulated to promote cell proliferation and block apoptosis [26]. Therefore, our gene analysis results imply that *H. pylori* may have promoted β-catenin oncogenic signaling via downregulation of its negative regulators and that ASTX treatment prevented the *H. pylori*-induced decline in Bambi and Smad4 expression.

APC, adenomatous polyposis coli; ATF3, activating transcription factor 3; Bambi, bone morphogenetic protein (BMP) and activating membrane-bound inhibitor; CK1, casein kinase 1; EGFR, epidermal growth factor receptor; FOSL1, Fos-like 1; GSK3β, glycogen synthase kinase β; KLFs, Kruppel-like factors; LRP, lipoprotein-related protein; MAPK, mitogen-activated protein kinase; SKP2, S-phase kinase-associated protein 2; Smad4, SMAD family member 4; Smox, spermine oxidase; TGF-β, transforming growth factor-β; β-Trcp, β-transducin repeat-containing protein; PKD2, polycystin2; PI3K, phosphoinositide 3-kinase.

**Figure 4:**
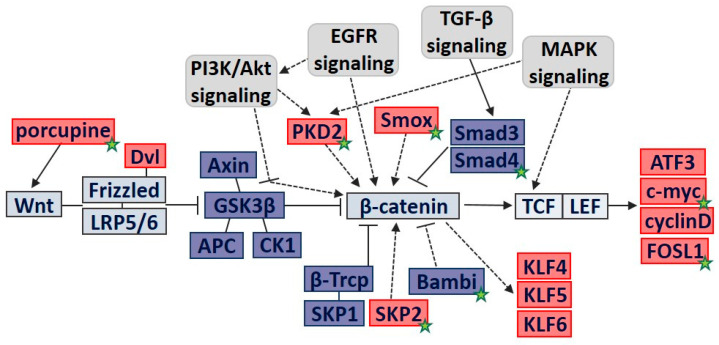
Schematic modeling of Wnt/β-catenin signaling network in *H. pylori*-infected AGS cells. Positive mediators in Wnt/β-catenin signaling are in red boxes and negative regulators are in blue boxes. Speculated molecular interactions are presented with dashed arrows. The DEG-encoded proteins that were differentially expressed by *H. pylori* and ASTX are marked with a star.

Using computational modeling based on the Wnt/β-catenin pathway of the KEGG database and relevant research references, a schematic outline of β-catenin signaling in gastric epithelial AGS cells is presented in Figure 4. When Wnt ligands are secreted, which depends on the activity of porcupine, Wnt protein binds to its Frizzled-lipoprotein-related protein (LRP) receptor. Activated Wnt receptor complex blocks the β-catenin destruction complex (Axin, adenomatous polyposis coli gene (APC), glycogen synthase kinase β (GSK3β), casein kinase 1 (CK1)) from phosphorylating β-catenin, which in turn frees β-catenin from proteasomal degradation by ubiquitin ligase β-Trcp. β-Catenin accumulates and translocates into the nucleus to bind TCF/LEF, and it induces expression of its target genes, such as activating transcription factor 3 (ATF3), c-myc, cyclin D, and Fos-like 1 (FOSL1).

Spermine oxidase (Smox) has been indicated in *H. pylori*-induced gastric carcinogenesis, which may be mediated by β-catenin. SMAD family member 4 (Smad4) and bone morphogenetic protein and activating membrane-bound inhibitor (Bambi), associated with the transforming growth factor-β (TGF-β) signaling pathway, may inhibit β-catenin signaling in *H. pylori*-infected AGS cells.

Polycystin2 (PKD2), S-phase kinase-associated protein 2 (SKP2), and Kruppel-like factors (KLFs) are involved in deregulation of cell growth and proliferation. Epidermal growth factor receptor (EGFR) or phosphoinositide 3-kinase (PI3K) signaling pathways can affect β-catenin-induced cell proliferation via regulators such as PKD2, SKP2, and KLFs. Mitogen-activated protein kinase (MAPK) signaling activates PKD2 and β-catenin/TCF/LEF binding to induce cell proliferation.

In summary, expression of Wnt protein activators such as porcupine activates the Frizzled/LRP receptor to sequester the destruction complex of Axin, APC, and GSK3β. *H. pylori* stimulation alters Bambi, PDK2, SKP2, Smox, and Smad4 to fully activate β-catenin signaling. Activated β-catenin signaling promotes cell proliferation via effector genes such as ATF3, FOSL1, and c-myc.

### 3.4. PCR Validation of Key Regulatory Genes Involved in H. pylori-Induced β-catenin Pathway

**Figure 5:**
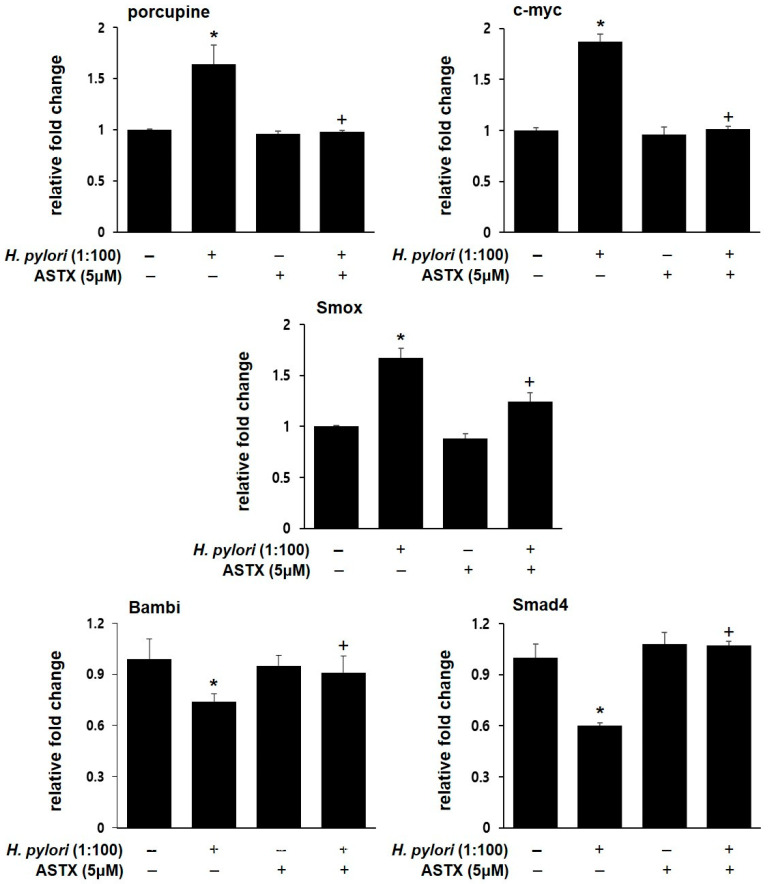
Changes in the mRNA expression of porcupine, c-myc, Smox, Bambi, and Smad4 of *H. pylori*-infected AGS cells with or without ASTX pretreatment. AGS cells were pretreated with vehicle (DMSO) or 5 μM ASTX for 3 h and then incubated with *H. pylori* at a cellular ratio of 100:1 for 4 h. Real-time PCR assay was performed to measure the relative amounts of porcupine, c-myc, Smox, Bambi, and Smad4 mRNA in AGS cells. mRNA levels were normalized to that of β-actin. * *p* < 0.05 vs. uninfected cells; + *p* < 0.05 vs. *H. pylori*-infected cells without ASTX pretreatment.

To verify the transcriptional expression patterns of the DEGs, real-time PCR for the representative genes was performed. Figure 5 shows that *H. pylori* infection increased the mRNA levels of porcupine, c-myc, and Smox, but downregulated Bambi and Smad4 expression. In contrast, ASTX pretreatment protected AGS cells against *H. pylori*-induced changes in gene expression. However, ASTX treatment alone had little or no effect on the mRNA levels.

## 4. Discussion

*H. pylori* infection of the stomach lining promotes gastric carcinogenesis by promoting inflammatory responses that lead to increased cell proliferation and migration. *H. pylori* secretes virulence factors such as VacA and Cag A to promote chronic inflammation, dysregulation of early immune responses in host cells, and ectopic host cell proliferation and apoptosis, as well as disruption of cell junctions and cell polarity [5]. Cellular signaling transduction pathways, such as EGFR, NF-κB, MAPK, PI3K/Akt, and Wnt are the major modulators of *H. pylori*-induced gastric carcinogenesis [27]. Previously, we observed upregulated activation of the β-catenin pathway, which subsequently led to cell proliferation of *H. pylori*-infected AGS cells [10,18]. In our preliminary study, we verified the activation of β-catenin, and we presumed that ASTX could inhibit *H. pylori*-induced β-catenin signaling.

In this study, RNA-Seq analysis was performed to explore the regulatory modulators of *H. pylori*-induced gastric oncogenesis and the possible effect of ASTX on *H. pylori*-induced oncogenic malignancies. In particular, GO annotation and molecular pathway analysis identified that DEGs in *H. pylori*-infected cells were notably associated with the Wnt/β-catenin signaling pathway. Among the various pathways involved in *H. pylori*-induced oncogenic transformation, Wnt/β-catenin signaling is the most extensively studied pathway that is directly correlated with aberrant epithelial proliferation [28]. ASTX treatment counter-regulated approximately half of the DEGs in *H. pylori*-infected cells, notably the genes associated with cancer pathways. Previous studies have demonstrated that ASTX effectively inhibits the proliferation of carcinoma cells by suppressing the NF-κB and Wnt/β-catenin pathways [29,30].

RNA-Seq analysis revealed that *H. pylori* induced overexpression of Fos-like 1, c-myc, TGF-β1, Smox, and porcupine. Specifically, activation of β-catenin led to elevated Fos-like 1 and c-myc expression, which collectively promotes cell proliferation and survival. Porcupine, which activates Wnt proteins, leads to elevated proliferation, migration, and invasion of gastric cancer cells via β-catenin signaling activation [31]. Our RNA-Seq results further revealed that ASTX treatment significantly repressed the expression of Fos-like 1, c-myc, and porcupine, which was validated using PCR analysis.

Smox, which catabolizes the polyamine spermine into spermidine and H_2_O_2_, has been extensively studied for its involvement in the development of cancer [32,33]. *H. pylori* infection elevates Smox expression in gastric epithelial cells, thereby inducing oxidative DNA damage and gastric dysplasia [34,35]. Further study showed that *H. pylori*-induced β-catenin accumulation was not observed in Smox–/– mice. Administration of Smox inhibitor not only repressed β-catenin activation but also blocked the expression of β-catenin target genes, indicating that Smox promotes *H. pylori*-induced carcinogenesis via β-catenin signaling. [21] Our results indicate that ASTX significantly inhibited *H. pylori*-induced Smox expression.

ASTX treatment reversed the *H. pylori*-induced downregulation of other genes, including Bambi and Smad4. Bambi was originally known to be a positive regulator of the oncogenic β-catenin pathway [36]. β-catenin stimulates Bambi transcription to inhibit growth-suppressive TGF-β/Smad signaling. However, surprisingly, Bambi expression was decreased in *H. pylori*-infected AGS cells. A previous study suggested a tumor-suppressive role of Bambi in hepatitis B virus-induced hepatocarcinoma [37]. Induction of Bambi expression suppressed β-catenin activation, TGF-β1 expression, and reduced growth and proliferation of hepatocarcinoma cells and tumor mass formation in vivo [37]. These findings concur with our observations of reduced Bambi expression and increased TGF-β1 expression in *H. pylori*-infected AGS cells.

Our study showed that ASTX treatment could significantly reverse *H. pylori*-induced downregulation of Smad4, which functions as a tumor suppressor in gastric epithelial AGS cells [38]. Inhibition of Smad4 enhanced cell proliferation, viability, and migration, which leads to the development of gastric cancer. By contrast, Smad4 expression abrogated cell proliferation and survival via β-catenin inactivation and transcriptional reduction in β-catenin-dependent genes such as cyclin D1, CD44, and c-MET. [39]

The current study has limitations due to lack of biological replicates for each treatment condition. Only one library per each group was prepared for this analysis, although it is recommended to have three technical replicates for each group to avoid false positives. Nevertheless, numerous qualitative PCR analyses on the molecular pathway of interest were performed before and after sequencing. The RNA-Seq analysis results from this study could be used as an exploratory analysis in finding transcriptomic patterns to be further investigated.

In conclusion, ASTX treatment is a potential therapeutic agent to suppress *H. pylori*-induced activation of the oncogenic Wnt/β-catenin pathway in gastric epithelial cells. *H. pylori* infection increased the expression of porcupine and Smox and downregulated the negative modulators Bambi and Smad4 in the β-catenin signaling pathway. ASTX treatment successfully blocked *H. pylori*-induced disruption of the signaling mediators porcupine, Smox, Bambi, and Smad4, as well as the expression of β-catenin effectors such as Fos-like 1 and c-myc.

We previously performed a similar RNA-Seq experiment on ASTX and *H. pylori* infection [19]. In the previous study we examined the short-term effect (1 h) of *H. pylori* infection (at cellular ratio of 50:1), whereas the current study was aimed for the intermediate stage (4 h), leading to cell proliferation observed in long-term infection (at cellular ratio of 100:1). Although the *H. pylori* infection ratio and exposure time differed, ASTX was administered under the same experimental conditions. Taken together, it can be suggested that ASTX clearly has an inhibitory effect on *H. pylori*-induced transcriptomic dysregulation in general. The anti-cancer effect of ASTX is not only based on modifications of cellular signaling pathways but also involves its epigenetic modulations as well [40,41]. Further studies are required in the future to elucidate how ASTX affects gene expressions in *H. pylori*-induced oncogenesis.

## Figures and Tables

**Table 1:** Expression levels of differentially expressed genes (DEGs) involved in the Wnt/β-catenin pathway. The expression level of each gene is reported as the read count normalized to the log2 value. For fold change values, red and blue background color was used to visualize increased and decreased gene expressions respectively, with the intensity of color representing the magnitude of change.

Gene Symbol	Fold Change		Gene Expression Value				Encoded Protein
	HP/ None	ASTX + HP/HP	Normalized Data (Log2)				
			None	HP	ASTX	ASTX + HP	
*BAMBI*	0.543	1.537	6.563	5.682	6.488	6.302	BMP and activin membrane-bound inhibitor
*FOSL1*	2.107	0.669	4.050	5.125	4.603	4.513	Fos-like 1
*TSPAN31*	1.517	0.513	3.900	4.501	4.352	3.537	tetraspanin 31
*E2F7*	0.640	1.635	5.146	4.501	5.185	5.210	E2F transcription factor 7
*ATXN1L*	0.630	1.514	4.629	3.964	4.423	4.562	ataxin 1 like
*SMAD4*	0.661	1.437	4.853	4.215	4.663	4.838	SMAD family member 4
*ZNF675*	0.642	1.538	4.340	3.700	4.848	4.321	zinc finger protein 675
*SERTAD3*	1.637	0.623	3.323	4.034	3.166	3.353	SERTA domain-containing protein 3
*TGFB1*	1.580	0.717	3.464	4.123	3.795	3.482	transforming growth factor-β1
*SMOX*	1.537	0.706	4.665	5.208	4.985	4.640	spermine oxidase
*PKD2*	0.585	1.680	4.809	4.034	4.764	4.783	polycystin 2
*SKP2*	0.548	1.524	6.606	5.739	6.205	6.347	S-phase kinase-associated protein 2
*MYC*	1.606	0.665	4.819	5.502	5.058	5.096	c-myc
*PORCN*	1.648	0.626	3.797	4.518	3.816	3.956	porcupine

## Data Availability

Data generated or analyzed during this study are included within the published article. Expression value of each gene is included in Table 1.

## References

[1] Thrift A.P., El-Serag H.B. (2020). Burden of gastric cancer. Clin. Gastroenterol. Hepatol..

[2] Ghotaslou R., Leylabadlo H.E., Nasiri M.J., Dabiri H., Hashemi A. (2018). Risk of gastric cancer in association with Helicobacter pylori different virulence factors: A systematic review and meta-analysis. Microb. Pathog..

[3] Díaz P., Valenzuela Valderrama M., Bravo J., Quest A.F. (2018). Helicobacter pylori and gastric cancer: Adaptive cellular mechanisms involved in disease progression. Front. Microbiol..

[4] Ding S.Z., Goldberg J.B., Hatakeyama M. (2010). Helicobacter pylori infection, oncogenic pathways and epigenetic mechanisms in gastric carcinogenesis. Future Oncol..

[5] Polk D.B., Peek R.M. (2010). Helicobacter pylori: Gastric cancer and beyond. Nat. Rev. Cancer.

[6] Clevers H., Nusse R. (2012). Wnt/β-catenin signaling and disease. Cell.

[7] Li V.S., Ng S.S., Boersema P.J., Low T.Y., Karthaus W.R., Gerlach J.P., Mohammed S., Heck A.J., Maurice M.M., Mahmoudi T. (2012). Wnt signaling through inhibition of β-catenin degradation in an intact Axin1 complex. Cell.

[8] Song X., Xin N., Wang W., Zhao C. (2015). Wnt/β-catenin, an oncogenic pathway targeted by H.pylori in gastric carcinogenesis. Oncotarget.

[9] Han H., Lim J.W., Kim H. (2020). Astaxanthin inhibits Helicobacter pylori-induced inflammatory and oncogenic responses in gastric mucosal tissues of mice. J. Cancer Prev..

[10] Kim D., Lim J.W., Kim H. (2019). β-carotene inhibits expression of c-myc and cyclin E in Helicobacter pylori-infected gastric epithelial cells. J. Cancer Prev..

[11] Ambati R.R., Phang S.M., Ravi S., Aswathanarayana R.G. (2014). Astaxanthin: Sources, extraction, stability, biological activities and its commercial applications—A review. Mar. Drugs.

[12] Hussein G., Sankawa U., Goto H., Matsumoto K., Watanabe H. (2006). Astaxanthin, a carotenoid with potential in human health and nutrition. J. Nat. Prod..

[13] Lim K.C., Yusoff F.M., Shariff M., Kamarudin M.S. (2018). Astaxanthin as feed supplement in aquatic animals. Rev. Aquac..

[14] Kim J.H., Park J.J., Lee B.J., Joo M.K., Chun H.J., Lee S.W., Bak Y.T. (2016). Astaxanthin inhibits proliferation of human gastric cancer cell lines by interrupting cell cycle progression. Gut Liver.

[15] Su X.Z., Chen R., Wang C.B., Ouyang X.L., Jiang Y., Zhu M.Y. (2019). Astaxanthin combine with human serum albumin to abrogate cell proliferation, migration, and drug-resistant in human ovarian carcinoma SKOV3 cells. Anti-Cancer Agents Med. Chem..

[16] McCall B., McPartland C.K., Moore R., Frank-Kamenetskii A., Booth B.W. (2018). Effects of astaxanthin on the proliferation and migration of breast cancer cells in vitro. Antioxidants.

[17] Kim S.H., Lim J.W., Kim H. (2018). Astaxanthin Inhibits Mitochondrial dysfunction and interleukin-8 expression in Helicobacter pylori-infected gastric epithelial cells. Nutrients.

[18] Park B., Lim J.W., Kim H. (2019). Lycopene treatment inhibits activation of Jak1/Stat3 and Wnt/β-catenin signaling and attenuates hyperproliferation in gastric epithelial cells. Nutr. Res..

[19] Kim S.H., Kim H. (2020). Transcriptome Analysis of the Inhibitory Effect of Astaxanthin on Helicobacter pylori-Induced Gastric Carcinoma Cell Motility. Mar. Drugs.

[20] Katoh M. (2018). Multi-layered prevention and treatment of chronic inflammation, organ fibrosis and cancer associated with canonical WNT/β-catenin signaling activation. Int. J. Mol. Med..

[21] Sierra J.C., Piazuelo M.B., Luis P.B., Barry D.P., Allaman M.M., Asim M., Sebrell T.A., Finley J.L., Rose K.L., Hill S. (2020). Spermine oxidase mediates Helicobacter pylori-induced gastric inflammation, DNA damage, and carcinogenic signaling. Oncogene.

[22] Liu K., Song X., Ma H., Liu L., Wen X., Yu J., Wang L., Hu S. (2014). Knockdown of BAMBI inhibits β-catenin and transforming growth factor β to suppress metastasis of gastric cancer cells. Mol. Med. Rep..

[23] Khin S.S., Kitazawa R., Win N., Aye T.T., Mori K., Kondo T., Kitazawa S. (2009). BAMBI gene is epigenetically silenced in subset of high-grade bladder cancer. Int. J. Cancer.

[24] Marwitz S., Depner S., Dvornikov D., Merkle R., Szczygieł M., Müller-Decker K., Lucarelli P., Wäsch M., Mairbäurl H., Rabe K.F. (2016). Downregulation of the TGFβ pseudoreceptor BAMBI in non–small cell lung cancer enhances TGFβ signaling and invasion. Cancer Res..

[25] Powell S.M., Harper J.C., Hamilton S.R., Robinson C.R., Cummings O.W. (1997). Inactivation of Smad4 in gastric carcinomas. Cancer Res..

[26] Xiao B., Zhu E.D., Li N., Lu D.S., Li W., Li B.S., Zhao Y.L., Mao X.H., Guo G., Yu P.W. (2012). Increased miR-146a in gastric cancer directly targets SMAD4 and is involved in modulating cell proliferation and apoptosis. Oncol. Rep..

[27] Yousefi B., Mohammadlou M., Abdollahi M., Salek Farrokhi A., Karbalaei M., Keikha M., Kokhaei P., Valizadeh S., Rezaiemanesh A., Arabkari V. (2019). Epigenetic changes in gastric cancer induction by Helicobacter pylori. J. Cell. Physiol..

[28] Liu Z., Sun R., Zhang X., Qiu B., Chen T., Li Z., Xu Y., Zhang Z. (2019). Transcription factor 7 promotes the progression of perihilar cholangiocarcinoma by inducing the transcription of c-Myc and FOS-like antigen 1. EBioMedicine.

[29] Kavitha K., Kowshik J., Kishore T.K.K., Baba A.B., Nagini S. (2013). Astaxanthin inhibits NF-κB and Wnt/β-catenin signaling pathways via inactivation of Erk/MAPK and PI3K/Akt to induce intrinsic apoptosis in a hamster model of oral cancer. Biochim. Biophys. Acta.

[30] Li J., Dai W., Xia Y., Chen K., Li S., Liu T., Zhang R., Wang J., Lu W., Zhou Y. (2015). Astaxanthin inhibits proliferation and induces apoptosis of human hepatocellular carcinoma cells via Inhibition of NF-κB P65 and Wnt/β-catenin in vitro. Mar. Drugs.

[31] Mo M.L., Li M.R., Chen Z., Liu X.W., Sheng Q., Zhou H.M. (2013). Inhibition of the Wnt palmitoyltransferase porcupine suppresses cell growth and downregulates the Wnt/β-catenin pathway in gastric cancer. Oncol. Lett..

[32] Casero R.A., Stewart T.M., Pegg A.E. (2018). Polyamine metabolism and cancer: Treatments, challenges and opportunities. Nat. Rev. Cancer.

[33] Goodwin A.C., Jadallah S., Toubaji A., Lecksell K., Hicks J.L., Kowalski J., Bova G.S., Marzo A.M., Netto G.J., Casero R.A. (2008). Increased spermine oxidase expression in human prostate cancer and prostatic intraepithelial neoplasia tissues. Prostate.

[34] Xu H., Chaturvedi R., Cheng Y., Bussiere F.I., Asim M., Yao M.D., Potosky D., Meltzer S.J., Rhee J.G., Kim S.S. (2004). Spermine oxidation induced by Helicobacter pylori results in apoptosis and DNA damage: Implications for gastric carcinogenesis. Cancer Res..

[35] Chaturvedi R., Asim M., Romero–Gallo J., Barry D.P., Hoge S., De Sablet T., Delgado A.G., Wroblewski L.E., Piazuelo M.B., Yan F. (2011). Spermine oxidase mediates the gastric cancer risk associated with Helicobacter pylori CagA. Gastroenterology.

[36] Sekiya T., Adachi S., Kohu K., Yamada T., Higuchi O., Furukawa Y., Nakamura Y., Nakamura T., Tashiro K., Kuhara S. (2004). Identification of BMP and activin membrane-bound inhibitor (BAMBI), an inhibitor of transforming growth factor-β signaling, as a target of the β-catenin pathway in colorectal tumor cells. J. Biol. Chem..

[37] Lee S., Lee M.J., Zhang J., Yu G.R., Kim D.G. (2016). C-terminal-truncated HBV X promotes hepato-oncogenesis through inhibition of tumor-suppressive β-catenin/BAMBI signaling. Exp. Mol. Med..

[38] Wang L.H., Kim S.H., Lee J.H., Choi Y.L., Kim Y.C., Park T.S., Hong Y.C., Wu C.F., Shin Y.K. (2007). Inactivation of SMAD4 tumor suppressor gene during gastric carcinoma progression. Clin. Cancer Res..

[39] Sun G.L., Li Z., Wang W.Z., Chen Z., Zhang L., Li Q., Wei S., Li B.W., Xu J.H., Chen L. (2018). miR-324-3p promotes gastric cancer development by activating Smad4-mediated Wnt/beta-catenin signaling pathway. J. Gastroenterol..

[40] Faraone I., Sinisgalli C., Ostuni A., Armentano M.F., Carmosino M., Milella L., Russo D., Labanca F., Khan H. (2020). Astaxanthin anticancer effects are mediated through multiple molecular mechanisms: A systematic review. Pharmacol. Res..

[41] El Omari N., Bakha M., Imtara H., Guaouguaoua F.E., Balahbib A., Zengin G., Bouyahya A. (2021). Anticancer mechanisms of phytochemical compounds: Focusing on epigenetic targets. Environ. Sci. Pollut. Res..

